# Enteroviral Infection: The Forgotten Link to Amyotrophic Lateral Sclerosis?

**DOI:** 10.3389/fnmol.2018.00063

**Published:** 2018-03-12

**Authors:** Yuan Chao Xue, Ralph Feuer, Neil Cashman, Honglin Luo

**Affiliations:** ^1^Centre for Heart and Lung Innovation, University of British Columbia, Vancouver, BC, Canada; ^2^Department of Pathology and Laboratory Medicine, University of British Columbia, Vancouver, BC, Canada; ^3^The Integrated Regenerative Research Institute at San Diego State University, San Diego, CA, United States; ^4^Djavad Mowafaghian Centre for Brain Health, University of British Columbia, Vancouver, BC, Canada

**Keywords:** amyotrophic lateral sclerosis, enterovirus, TDP-43 pathology, nucleocytoplasmic trafficking, RNA metabolism, autophagy, neuroinflammation

## Abstract

Amyotrophic lateral sclerosis (ALS) is a devastating neurodegenerative disease that primarily attacks motor neurons in the brain and spinal cord, leading to progressive paralysis and ultimately death. Currently there is no effective therapy. The majority of ALS cases are sporadic, with no known family history; unfortunately the etiology remains largely unknown. Contribution of Enteroviruses (EVs), a family of positive-stranded RNA viruses including poliovirus, coxsackievirus, echovirus, enterovirus-A71 and enterovirus-D68, to the development of ALS has been suspected as they can target motor neurons, and patients with prior poliomyelitis show a higher risk of motor neuron disease. Multiple efforts have been made to detect enteroviral genome in ALS patient tissues over the past two decades; however the clinical data are controversial and a causal relationship has not yet been established. Recent evidence from *in vitro* and animal studies suggests that enterovirus-induced pathology remarkably resembles the cellular and molecular phenotype of ALS, indicating a possible link between enteroviral infection and ALS pathogenesis. In this review, we summarize the nature of enteroviral infection, including route of infection, cells targeted, and viral persistence within the central nervous system (CNS). We review the molecular mechanisms underlying viral infection and highlight the similarity between viral pathogenesis and the molecular and pathological features of ALS, and finally, discuss the potential role of enteroviral infection in frontotemporal dementia (FTD), a disease that shares common clinical, genetic, and pathological features with ALS, and the significance of anti-viral therapy as an option for the treatment of ALS.

## Introduction

Amyotrophic lateral sclerosis (ALS) is a fatal neurodegenerative disease marked by progressive degeneration of both upper and lower motor neurons, resulting in paralysis and eventual death within 3–5 years after diagnosis (Brown and Al-Chalabi, 2017; Hardiman et al., 2017; van Es et al., 2017). Despite primarily a motor neuron disease, nearly 50% of ALS patients show cognitive/behavioral impairments, suggesting an involvement of non-motor systems in ALS pathogenesis (Hardiman et al., 2017). The overall prevalence of ALS in Europe and North America is estimated at ~3–5 cases per 100,000 people and increases with age (Brown and Al-Chalabi, 2017). The average age of ALS onset is between 55 and 65, with the cumulative lifetime risk being higher for men (1:350) than for women (1:400; van Es et al., 2017). There is currently no cure for ALS. Riluzole (a glutamate release inhibitor) and edaravone (a free-radical scavenger) are the only two FDA-approved drugs for the treatment of ALS. However, their benefits are modest by only delaying disease progression and prolonging survival for 2–3 months (Martinez et al., 2017). Many other therapeutic options have been investigated over the past two decades, but their clinical effectiveness has not been proven (Katyal and Govindarajan, 2017; Martinez et al., 2017).

ALS can be genetically inherited or occur sporadically in individuals without any apparent family history. Since the identification of *SOD1* as a causative gene of ALS, significant progress has been made in unravelling the genetics causes of ALS. Over the past two decades, ~30 genes have been identified to be highly associated with ALS (Al-Chalabi et al., 2017; Ito et al., 2017; Chia et al., 2018). These genes encode proteins that are involved in the maintenance of protein homeostasis and vesicle trafficking (e.g., *SQSTM1/p62*, *TBK1*, *SOD1*, *VCP*, *CHMP2B*, *OPTN*, *UBQLN2 and C9ORF72*), RNA processing (e.g., *TARDBP*, *FUS*, *TIA1*, *HNRNPA1*, *C9ORF72* and *MATR3*), and regulation of cytoskeletal integrity and axonal transport (e.g., *PFN1*, *DCTN1*, *SOD1*, and *TUBA4A*, Al-Chalabi et al., 2017; Mackenzie et al., 2017; Chia et al., 2018). Unlike familial ALS (FALS), the cause(s) of sporadic ALS (SALS), which accounts for the majority of ALS cases (90%–95%), remain(s) largely unclear. A “multistep model” of gene-environment interaction requiring both genetic mutations and environmental risk factors has been proposed as a mechanism triggering the onset and progression of SALS (Al-Chalabi et al., 2014). Several environmental risk factors have been studied, including viral exposure, physical activity, smoking, heavy metals, pesticides and chemicals, military service, and electric shock (Yu and Pamphlett, 2017). However, a definitive relationship between these factors and ALS has yet to be established. This review will focus on the possible contribution of viral infection, in particular, enteroviral infection in the pathogenesis of ALS.

## Enteroviruses and Neurological Disorders

Enteroviruses (EVs) are a group of single, positive-stranded RNA viruses of the *Picornaviridae* family that include poliovirus, coxsackievirus, echovirus and enterovirus, with the latter (specifically EV-A71 and EV-D68) emerging as the causative agents of the recent large epidemics across the Asia-Pacific and North American region, respectively (Huang and Shih, 2015; Anastasina et al., 2017). Although EVs commonly cause asymptomatic infection, sometimes they are associated with severe diseases, including neurological complications. EVs have a high tropism for the central nervous system (CNS) and account for various neurological disorders, such as poliomyelitis, aseptic meningitis, encephalitis and non-polio flaccid paralysis, particularly in infants and children (Rhoades et al., 2011; Huang and Shih, 2015). Since the successful campaign of the poliovirus vaccination, neurological diseases caused by non-polio EVs have been increasingly reported. For example, acute flaccid paralysis was frequently observed among patients with EV-A71, echovirus and coxsackievirus infection (Suresh et al., 2018). In addition, epidemiological studies from the recent EV-D68 outbreaks reveal a strong relationship between EV-D68 infection and increased incidence of acute flaccid myelitis (Greninger et al., 2015; Messacar et al., 2016).

While the majority of the EVs are transmitted through the fecal-oral route and replicate in the gastrointestinal tract, some EVs (e.g., EV-D68) can cause respiratory infection and spread *via* respiratory secretion. Available evidence suggests that EVs can invade the CNS from these primary infection sites through three main mechanisms: (1) retrograde axonal transport—both poliovirus and EV-A71 can infect the peripheral nerve and gain access into the CNS via retrograde axonal transport and trans-synaptic spread (Gromeier and Wimmer, 1998; Chen et al., 2007); (2) blood-brain barrier (BBB) penetration—during viremia, poliovirus in the blood can directly cross the BBB through disrupted tight junctions that are likely induced by inflammation independently of viral receptor (Yang et al., 1997), and/or *via* transferrin receptor 1-mediated direct transmission (Mizutani et al., 2016); and (3) “Trojan-horse” invasion—EVs, such as poliovirus (Freistadt and Eberle, 1996), EV-A71 (Lin et al., 2009b) and coxsackievirus (Tabor-Godwin et al., 2010), can also invade the CNS through virus-infected immune cells, including macrophage/monocytes, dendritic cells, lymphocytes and nesting^+^ myeloid cells, which act as carriers to deliver virus into the CNS. EVs likely utilize one or multiple routes of entry into the CNS.

Within the CNS, many cell types can be targeted by the EVs. It is well documented that poliovirus infects and replicates in motor neurons within the anterior horns of the spinal cord, leading to poliomyelitis (Nagata et al., 2004). Motor neurons in the spinal cord and brainstem are also highly susceptible to EV-71 (Ong and Wong, 2015; Too et al., 2016). More recently, it was reported that mice infected with EV-D68 isolates from the 2014 outbreak develop limb paralysis, closely resembling human acute flaccid myelitis, due to infection of motor neurons in the anterior horns of spinal cord (Hixon et al., 2017). In addition to neuronal cells, especially motor neurons, astrocytes and oligodendrocytes are also permissive to poliovirus (Couderc et al., 2002), EV-A71 (Tung et al., 2010), and coxsackievirus B3 (CVB3; Zhang et al., 2013). Moreover, CVB3 and EV-A71 were found to preferentially target neural progenitor cells compared with differentiated neuronal cells, suggesting a mechanism of viral persistence and possible lasting neurological consequences (Feuer et al., 2005; Tsueng et al., 2011; Huang et al., 2014).

Although EVs are regarded as highly lytic viruses and EV-related diseases are commonly resulted from acute infection, EVs, such as poliovirus (Julien et al., 1999), EV-A71 (Han et al., 2010), and coxsackievirus (Feuer et al., 2009), can persist in various tissues, including the CNS. Glial cells and neuronal progenitor cells were reported to be the sites of CVB3 persistence (Feuer et al., 2009; Zhang et al., 2013). Multiple viral and host factors, including viral receptors, viral mutations, viral evasion of host immune response, and host translation machinery, participate in establishing a persistent EV infection (Rhoades et al., 2011; Huang and Shih, 2015). Latent EVs might be reactivated years later, either spontaneously or in response to exogenous stimulations, such as local trauma (Andréoletti et al., 2000; Feuer et al., 2002). EV persistence in cardiomyocytes and pancreatic cells has been associated with chronic clinical conditions, such as dilated cardiomyopathy and type 1 diabetes, mainly through continuous inflammatory responses (Chapman and Kim, 2008; Oikarinen et al., 2012). However, the long-term impacts of EV infection within the CNS are largely unclear. Clinically, it is observed that polio survivors decades after the recovery from the acute paralytic poliomyelitis can develop post-poliomyelitis syndrome, a neurological disorder characterized by new and progressive muscular weakness, accompanied by the detection of defective viral particles in the cerebrospinal fluid of some patients (Dalakas, 1995), indicating a possible long-lasting effect of latent poliovirus infection. In addition, a murine model has shown that a neonatal CVB3 infection can have a chronic impact on neurogenesis and CNS development, further supporting a potential link between early subclinical infections and late neurological sequelae (Ruller et al., 2012).

## Viral Infection and ALS

Viral infection has long been suspected as an environmental risk factor and/or a causative pathogen for ALS. Over the past 30 years, many efforts have been made to explore the association of neurotropic viruses (Limongi and Baldelli, 2016), especially EVs, exogenous retroviruses such as human immunodeficiency virus (Verma and Berger, 2006) and human T cell leukemia virus (Araujo, 2015), and human endogenous retrovirus (HERV; Li et al., 2015), with ALS.

HERVs are the remnants of ancient retroviruses integrated into the human genome and normally inactivate, but can be re-activated under physiological and pathological stresses (Li et al., 2015). Despite unsuccessful attempts to find evidence of exogenous retroviruses in ALS patient tissues (McCormick et al., 2008), several studies have reported the detection of enhanced gene expression of HERV-K and reverse transcriptase activity in the blood (Andrews et al., 2000; Steele et al., 2005; MacGowan et al., 2007) and brain tissues (Douville et al., 2011; Li et al., 2015) of ALS patients. More notably, transgenic mice expressing HERV-K in the neurons develop progressive motor neuron dysfunction similar to human ALS phenotype (Li et al., 2015), suggesting a potential viral etiology of ALS. Further studies are underway to explore the mechanism by which HERV-K causes neuropathology and to test existing anti-retroviral drugs and develop novel anti-HERV inhibitors for the treatment of retrovirus-associated ALS (Bowen et al., 2016; Tyagi et al., 2017).

A potential role of EVs in ALS has been proposed for decades due to their ability to target motor neurons and the development of the ALS-like post-poliomyelitis-syndrome (Ravits, 2005). Multiple clinical studies have been conducted to detect EVs in ALS patient tissues; however, the available data are controversial and inconclusive. Using RT-PCR, three studies reported a 60%–88% incidence of EV genome detection in spinal cord/brain of ALS patients, compared to 0%–14% in controls (Woodall et al., 1994; Berger et al., 2000; Giraud et al., 2001). Additionally, RT-PCR analysis of cerebrospinal fluid showed EV detection in 14.5% of 242 ALS patients and 7.6% of 354 controls (Vandenberghe et al., 2010). However, three additional studies failed to detect EV RNA in spinal cord/brain of either ALS patients or controls (Swanson et al., 1995; Walker et al., 2001; Nix et al., 2004). The discrepancies between these studies are likely due to methodological differences, such as the use of fresh vs. archived (or frozen vs. fixed) tissues, and differences in PCR primers/amplification methods, which can all affect the integrity of viral RNA and the sensitivity/specificity of viral genome detection. Moreover, the stage of the disease when samples are collected may also be critical for viral detection, as viruses may be detectable or active only in certain phases of the disease or in only a subset of patients. In addition, given the emerging “prion-like mechanism” in ALS pathogenesis (Grad et al., 2015), it is also possible that EV infection causes disease pathology (i.e., seeding of protein misfolding) focally during the acute phase of childhood infection, followed by the gradual propagation of misfolded proteins in other regions of the CNS, eventually leading to ALS onset in adulthood. In such case, an active viral infection may not be required for disease progression. Studies involved population-based retrospective cohort may be one way to “enrich” the chances of linking EV infection to later ALS. But the most common symptoms of EV infections are flu-like symptoms, which are often neglected until more disastrous consequences arise. Thus, the existing clinical EV infection data may not be an accurate reflection of the real infection prevalence. Overall, with these limitations, it would be very difficult to come up with a compelling strategy to firmly establish the connection between EV infection and ALS in humans. However, recent evidence from *in vitro* and animal studies prompts us to re-visit the longstanding controversial role of EVs in ALS from a different perspective.

## EV-Induced Molecular and Pathological Changes Associated With ALS Pathogenesis

**Figure 1:**
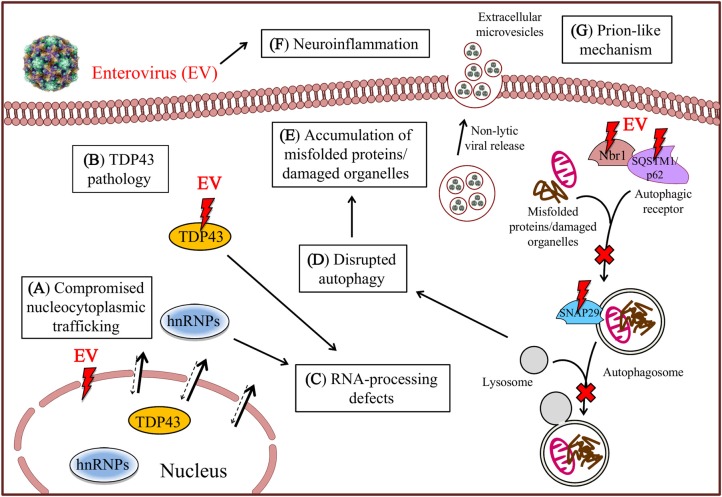
Molecular and pathological similarities between amyotrophic lateral sclerosis (ALS) and enteroviral infection. Enterovirus (EV) infection impairs nucleocytoplasmic trafficking **(A)** *via* EV proteinase-mediated proteolysis of nucleoporin proteins, resulting in mislocalization of TDP-43 and heterogeneous nuclear ribonucleoproteins (hnRNPs) from the nucleus to the cytoplasm, where they are further cleaved to induce TDP-43 pathology **(B)** and cause RNA-processing defects **(C)**. EV infection also results in the cleavage of several critical autophagic proteins, including SQSTM1/p62, Nbr1 and SNAP29, contributing to the disruption of the autophagic pathway **(D)** and consequent accumulation of misfolded proteins/damaged organelles **(E)**. Finally, subclinical EV infection induces chronic inflammatory reaction **(F)** and promotes non-lytic viral spread and prion-like mechanism through extracellular microvesicles **(G)**.

Emerging evidence from cell culture and mouse experiments reveals that EV infection produces hallmark cellular and molecular phenotypes of ALS, including RNA-processing defects, impaired nucleocytoplasmic transport, neuroinflammation, compromised protein quality control, and most strikingly, TDP-43 (transactive response DNA binding protein-43) pathology, supporting a potential link between EV infection and ALS pathogenesis (Figure 1).

### RNA-Processing Defects and TDP-43 Pathology

The significance of aberrant RNA metabolism in ALS is increasingly recognized since the discoveries of ALS-causing mutations in many genes that encode RNA-binding proteins (RBPs), including *TARDBP*, *FUS*, *HNRNPA1*, *MATR3*, *TAF15* and *TIA1* (Ito et al., 2017). More notably, cytoplasmic mislocalization, aggregation, and cleavage of TDP-43 (TDP-43 pathology) in motor neurons and glial cells have been found in ~97% of all ALS cases, and consequently regarded as a pathological hallmark of ALS (Neumann et al., 2006; Mackenzie et al., 2007). TDP-43, encoded by *TARDBP*, is a ubiquitously expressed DNA/RNA-binding protein and plays a critical role in regulating RNA metabolism (Lee et al., 2012). Mechanistically, both loss- and gain-of-function in TDP-43 pathology are believed to contribute to disease onset and progression (Lee et al., 2012). The replication of EVs, including poliovirus, EV-A71, and coxsackievirus, which takes place in the cytoplasm, relies heavily on the function of cellular proteins. Numerous RBPs, which are mostly nucleocytoplasmic shuttling proteins and localized predominantly to the nucleus, are detained in the cytoplasm during EV infection, and hijacked by EVs for support of viral translation and viral RNA replication (Lloyd, 2015). For example, EV-A71 infection causes redistribution of heterogeneous nuclear ribonucleoprotein (hnRNP) A1 from the nucleus to the cytoplasm, where it stimulates EV-A71 replication by facilitating internal ribosome entry site-mediated viral translation (Lin J. Y. et al., 2009; Tolbert et al., 2017). Most intriguingly, it was found that CVB3 infection results in cytoplasmic mislocalization and cleavage of TDP-43 *via* the action of CVB3-encoded proteinases, leading to RNA-processing deficits (Fung et al., 2015). Together, these findings suggest that EV infection shares a strikingly similar molecular feature in RNA metabolism with ALS.

### Compromised Nucleocytoplasmic Trafficking

Defects in nucleocytoplasmic shuttling have recently been identified as a central theme of ALS research, contributing significantly to the pathological hallmark of cytoplasmic mislocalization of RBPs (Boeynaems et al., 2016). Although the underlying mechanisms remain largely elusive, recent studies demonstrate that the G4C2 hexanucleotide repeat expansion mutation in the first intron of chromosome 9 open reading frame 72 (*C9orf72*) plays an important role in the dysfunction of nucleocytoplasmic transport (Freibaum et al., 2015; Jovicic et al., 2015; Xiao et al., 2015; Zhang et al., 2015). Mutation in *C9orf72* is the most common genetic cause of ALS, responsible for ~40% of FALS and 5%–10% of SALS (DeJesus-Hernandez et al., 2011; Renton et al., 2011). It was discovered that expression of G4C2 repeats impairs nucleocytoplasmic transport at the levels of transcribed G4C2 repeat RNA and translated dipeptide repeat proteins through interfering with the function of Ran GTPase-activating protein 1 and by disrupting the integrity of the nuclear pore complex (NPC; Freibaum et al., 2015; Jovicic et al., 2015; Xiao et al., 2015; Zhang et al., 2015). Likewise, compromised nucleocytoplasmic trafficking is a common mechanism for EV-induced pathology (Yarbrough et al., 2014). It was found that nucleoporin (Nup) proteins, Nup62, Nup98 and Nup153, key components of the NPC, are targeted by poliovirus-encoded proteinase 2A for degradation, resulting in the blockage of nuclear import (Gustin and Sarnow, 2001; Park et al., 2008, 2015). Through this strategy, EVs gain access to the otherwise predominantly nuclear proteins necessary for effective viral replication and inhibit host immune response by preventing nuclear transport of anti-viral signal molecules (Yarbrough et al., 2014). A recent finding that expression of CVB3 proteinase 2A alone is sufficient to induce cytoplasmic accumulation of TDP-43 indicates a possible overlapping mechanism linking EV to ALS (Fung et al., 2015).

### Neuroinflammation

The immune involvement in the development of ALS has been widely studied, with most focused on the activation of glial cells and astrocytes as such event would lead to the up-regulation of multiple pro-inflammatory cytokines, such as tumor necrosis factor-α, monocyte chemoattractant protein-1, cyclooxygenase-2, and interleukins (Sekizawa et al., 1998; Almer et al., 2001; Robertson et al., 2001). For viral infection, beyond direct damage, EVs could also stimulate immune-mediated injury by facilitating the production of pro-inflammatory cytokines, leukocyte infiltration and astrogliosis (Lin et al., 2003; Feuer et al., 2009; Rhoades et al., 2011; Ruller et al., 2012). It was shown that chronic CVB3 infection induces CNS microgliosis and astrogliosis in persistently infected mouse brains (Feuer et al., 2009). The presence of immune cells and cytokines within the CNS can worsen the virus-mediated neuropathology and the potential bystander damage caused by the subsequent T cell activation (Lin et al., 2009a). Furthermore, neuroinflammation initiated by microglia and astrocytes can trigger cell death by promoting the production of reactive oxygen species. Altogether, current evidence supports the possibility that chronic EV infection is able to induce late-onset CNS dysfunction by inducing inflammatory reactions.

### Defective Autophagy

In addition to RNA metabolism, ALS-causing mutations in genes, such as *SQSTM1*/*p62, OPTN, TBK1, VCP and UBQLN2*, are frequently related to protein quality control (Al-Chalabi et al., 2017; Chia et al., 2018). Autophagy deficits and consequent accumulation of misfolded proteins and large RNA clusters (e.g., RNA granules) are implicated in ALS pathogenesis (Cipolat Mis et al., 2016). Recent studies indicate that EVs can hijack the autophagic pathway to their own advantage. Autophagy is a dynamic process comprised of autophagosome formation and degradation following fusion with lysosome, called autophagic flux. Autophagic flux is inhibited upon EV infection, due at least in part to EV proteinase-mediated cleavage of synaptosomal-associated protein 29, a critical component of the soluble N-ethylmaleimide-sensitive factor activating protein receptor (SNARE) complex required for autophagosome-lysosome fusion (Corona et al., in press; Mohamud et al., in press). As a result of reduced autophagic flux, autophagosomes accumulate and favor viral growth by providing membrane scaffolds for viral assembly and replication (Shi and Luo, 2012). Inhibition of autophagic flux also leads to accumulation of protein aggregates, contributing to viral pathogenesis. Apart from bulk degradation, autophagy can selectively recycle misfolded proteins/damaged organelles, a process mediated by autophagic receptors, including sequestosome 1 (SQSTM1)/p62 and neighbor of BRCA1 (Nbr1), which target ubiquitinated proteins/organelles to autophagosomes for destruction (Shaid et al., 2013). EV infection leads to cleavage of SQSTM1 and/or Nbr1 *via* the proteolytic activity of EV proteinases, resulting in impaired clearance of protein aggregates (Shi et al., 2013, 2014). Collectively, current evidence suggests a novel molecular mechanism employed by EVs to promote viral replication and induce ALS-like viral pathogenesis.

### Prion-Like Mechanism

ALS is known to start focally and spread to other regions in a neuroanatomic fashion (Grad et al., 2015). Recent evidence suggests that both exosome-dependent and -independent mechanisms are involved in the transmission of misfolded proteins (Grad et al., 2014a,b). As non-enveloped viruses, EVs are traditionally suggested to only be able to exit the infected cell by causing it to rupture. However, it has become increasingly clear that this group of viruses, including poliovirus, CVB3, and EV-A71, can also spread between cells in a non-lytic fashion *via* extracellular microvesicles (EMVs), such as exosomes (Bird et al., 2014; Robinson et al., 2014; Chen et al., 2015; Too et al., 2016). In doing so, EVs acquire a membrane shield against host immune detection. It is thus conceivable that EV-induced secretion of EMVs represents a mechanism for the transmission of misfolded proteins (e.g., misfolded SOD1 and TDP-43) within the CNS during chronic infection.

## Conclusion

SALS is an idiopathic, fatal neurodegenerative disorder of the motor neuron system, with no effective treatment to date. An EV etiology of SALS has been proposed for decades; however, human viral interrogation studies show conflicting results. Emerging evidence has revealed that EV infection induces signature molecular features of ALS. This evidence, along with the earlier findings that EVs can establish a persistent infection in the CNS, suggests that chronic and persistent EV infection might be a causal/risk factor for ALS. Further investigations are needed to firmly establish the relationship, for example, by assessing whether subclinical EV infection can promote early onset and progression of ALS in normal mice or mice that are genetically susceptible to ALS.

Recent progress has led to a greater recognition of the significant clinical and genetic overlaps between ALS and frontotemporal dementia (FTD). In fact, ~13% of ALS patients also have FTD (Brown and Al-Chalabi, 2017; Hardiman et al., 2017; van Es et al., 2017). Mutations in the same set of genes, including *C9ORF72*, *TARDBP*, *FUS*, *TIA1* and *SQSTM1/p62*, that cause ALS, are also linked to FTD (Gao et al., 2017). In addition, ALS and FTD also share common neuropathological hallmarks and disease mechanisms. It is therefore reasonable to postulate that EV infection also plays a role in FTD. Finally, identification of EV as a novel causal/risk factor for SALS will offer a huge potential for future therapeutic interventions. During EV infection, the use of ribavirin, a general anti-RNA viral drug that is able to cross the BBB, long after the end of the acute infection can greatly reduce viral-mediated neuropathology (Ruller et al., 2012). Moreover, pleconaril, an anti-picornavirus drug with demonstrated efficacy against many EVs, including EV-D68 (Liu et al., 2015), has been shown to cross the BBB and limit multiple species of EV infection within the CNS (Schmidtke et al., 2009). Thus, it is anticipated that anti-viral therapy offers new hope for the treatment of ALS.

## Author Contributions

All authors contributed to the writing and discussion of this review article.

## Conflict of Interest Statement

The authors declare that the research was conducted in the absence of any commercial or financial relationships that could be construed as a potential conflict of interest.
